# Endocannabinoids and Immunity

**DOI:** 10.1089/can.2016.0002

**Published:** 2016-02-01

**Authors:** Valerio Chiurchiù

**Affiliations:** ^1^ School of Medicine and Center of Integrated Research, Campus Bio-Medico University of Rome, Rome, Italy.; ^2^ European Center for Brain Research (CERC), I.R.C.C.S. Santa Lucia Foundation, Rome, Italy.

**Keywords:** endocannabinoid, immunology, immunosuppression

## Abstract

Over the last 10 years, a great boost of knowledge accumulated on the immunomodulatory and anti-inflammatory properties of endocannabinoids (eCBs). In this scenario, these bioactive lipids, which are produced by most immune cells along with a set of receptors and enzymes that regulate their synthesis and degradation, act as secondary modulators and increase or decrease a plethora of immune functions. In this review, the manifold immunomodulatory effects of the main eCBs in different compartments of innate and adaptive immunity will be discussed, suggesting that they could be considered as master regulators of innate-adaptive immune axis and as potent immunoresolvents.

## Endocannabinoids and Their Metabolism in the Immune System

Endocannabinoids (eCBs) include a group of lipid mediators, the best characterized of which are *N*-arachidonoylethanolamine (mostly known as anandamide, AEA) and 2-arachidonoylglycerol (2-AG).^1,2^ Some other compounds have been proposed to belong to the eCB family, including 2-AG-ether (noladin ether) and *O*-arachidonoylethanolamine (virodhamine), as well as two additional *N*-acylethanolamines (NAEs), namely *N*-palmitoylethanolamine (PEA) and *N*-oleoylethanolamine (OEA).^1,2^ eCBs are synthesized and released on demand (if and when needed) from membrane phospholipids in response to physiological or pathological stimuli, although recent evidence has shown that they could be accumulated and stored by intracellular transporters and storage organelles/pools.^3^ It is now well established that all body districts and tissues produce eCBs as part of a homeostatic system that acts at almost every level of biological life, with the aim of controlling several physiopathological states and maintaining human health. Their role in the regulation of the immune system is probably the most flourishing and promising research field due to the increasing recognition of the eCBs signaling in several chronic inflammatory diseases.^1,4^ In particular, AEA and other NAEs are synthesized from phosphatidylethanolamine through a series of enzymatic steps that involve mainly the enzyme *N*-acyl-phosphatidylethanolamine-hydrolyzing phospholipase D (NAPE-PLD) as well as, alternatively, phospholipase A and lyso-PLD, α/β-hydrolase 4 (ABDH4) and glycerophosphodiesterase 1, or phosholipase C and protein tyrosine phosphatase type-22.^2^ On the contrary, 2-AG is synthesized from *sn*-1-acyl-2-arachidonoylglycerols (DAGs) through two Ca^2+^-sensitive *sn*-2-selective DAG lipases (DAGL-α and DAGL-β) or more rarely from 2-arachidonoylglycerol-3-phosphate (2-AG-3P).^2^ Once synthesized, eCBs bind to and functionally activate their target receptors, triggering various signaling pathways and causing several biological effects on different tissues. The main receptor targets for eCBs are type-1 (CB_1_) and type-2 (CB_2_) G protein-coupled cannabinoid receptors. CB_1_ is widely expressed in the nervous system mainly at the terminal ends of central and peripheral neurons and its presence has also been widely documented in periphery. CB_2_ is mainly expressed by cells of the immune system, where it is commonly associated with the regulation of different immune functions.^1,4^ Yet, CB_2_ can also be found in neurons and activated microglial cells and astrocytes in response to various insults, particularly in association with chronic inflammation of the nervous system.^5^ However, eCBs can bind to and activate other receptors, including the transient receptor potential vanilloid 1 (TRPV1) channel, peroxisome proliferator-activated receptor (PPAR) α and γ, and the orphan G protein-coupled receptor GPR55, all of them being also widely expressed in immune cells.^1,5,6^ For instance, PPARα and PPARγ mediate part of the eCB-induced immunomodulatory effects on several immune cells,^7,8^ and GPR55 was recently found to be specifically expressed on monocytes and natural killer (NK) cells and to mediate activatory properties.^9^ The inactivation of eCBs involves cellular uptake through a purported and still unidentified endocannabinoid membrane transporter and subsequent intracellular hydrolysis. AEA is mainly cleaved not only by fatty acid amide hydrolase (FAAH) into arachidonic acid and ethanolamine but also by another enzyme, *N*-acylethanolamine-hydrolyzing acid amidase (NAAA), which is mainly involved in the hydrolysis of PEA.^2^ 2-AG, instead, is cleaved into glycerol and arachidonic acid not only mainly by monoacylglycerol lipase (MAGL) but, in part, also by FAAH, α/β-hydrolase domain-containing protein 6 (ABHD6), and 12 (ABHD12).^2^ Furthermore, AEA and 2-AG can also be metabolized by cyclooxygenase-2 (COX-2), lipoxygenase (LOX) isozymes, and cytochrome P450, generating several oxidized compounds such as prostaglandin-ethanolamides and glyceryl esters, hydroxy-anandamides, and hydroxyeicosatetraenoyl-glycerols.^10^ As it will be discussed in detail in the next session, it is no surprise that the immunosuppressive effects of eCBs on immune cells are primarily mediated through CB_2_, whose expression is indeed higher than that of CB_1_.^1,4^

## Innate Immunity

### Monocytes/macrophages

**FIG. 1.**
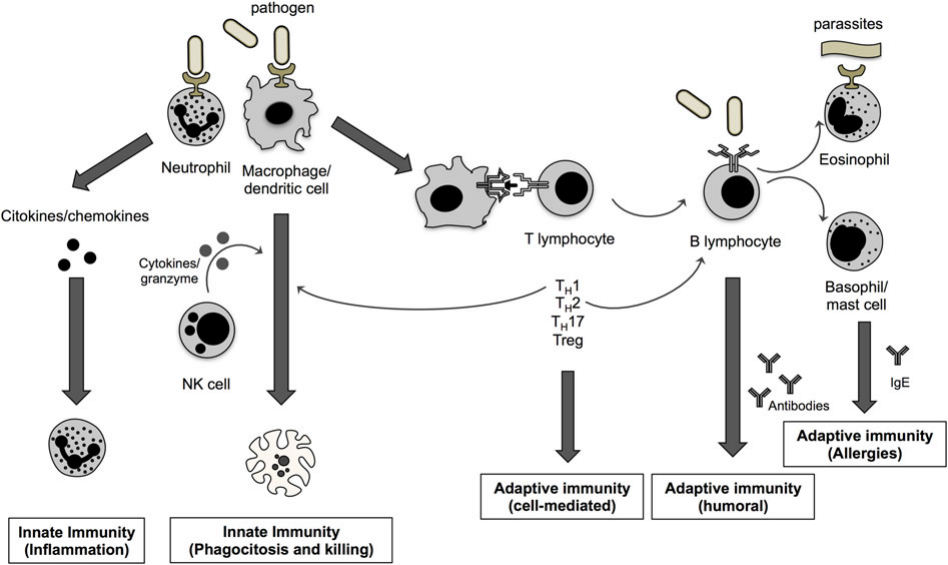
Schematic representation of the immune system and the functional interaction between immune cells.

Macrophages (and monocytes, their precursor cells) play an important role in innate immunity since they do not only clear apoptotic cells and pathogens but they also instruct other immune cells (Fig. 1). Monocytes/macrophages are highly plastic (they can change their functional phenotype depending on environmental cues) and reside in every tissue of the body, where they bear different names (i.e., Kupffer cells in the liver or microglia in the central nervous system),^11^ CB_1_ and CB_2_ receptors are highly expressed in both murine and human monocytes/macrophages and microglial cells regardless of cellular models.^1,12,13^

### Dendritic cells

Dendritic cells (DCs) are the most professional antigen-presenting cells, crucial in the development of antigen-specific T-cell responses (Fig. 1). They are present in those tissues that are in contact with the external environment, such as the skin (i.e., Langerhans cells) and the inner lining of several organs; they can also be found in peripheral blood (i.e., myeloid and plasmacytoid DCs).^14^ Despite their role in shaping the type and quality of immune responses due to their position at the crossroads between innate and adaptive immunity, very few studies have investigated eCB signaling in these cells, especially in humans.

### Neutrophils and NK cells

Neutrophils and NK cells are crucial elements of innate immunity and are both involved in host defense against cancer and antimicrobial responses (Fig. 1). Neutrophils are the first inflammatory cells to be recruited at the site of inflammation/injury and are the hallmark of acute inflammation. Although, neutrophils express very low levels of cannabinoid receptors, a great deal of information has been accumulated on the role of both AEA and 2-AG in human neutrophils, maybe due to their abundance in peripheral blood. On the contrary, knowledge on eCB signaling in NK cells, which are a type of cytotoxic lymphocytes that provide rapid responses against virally infected cells and cancer cells,^15,16^ is almost null, although these cells have been shown to express CB_1_, CB_2_, and GPR55, as well as to release high levels of AEA and 2-AG.^1^

### Eosinophils, basophils, and mast cells

These rare cell populations share similar appearance and function and are involved in allergy and anaphylaxis, as well as in wound healing and in defense against pathogens (Fig. 1). However, they differ, in that they arise from different cell lines and in that eosinophils and basophils are found in the blood, whereas mast cells are tissue resident (i.e., connective and mucosal tissue, nervous system).^17,18^ Furthermore, eosinophils play a major role in dealing with elimination of large parasites.^18^ As yet, no evidence has been reported on eCB signaling either on murine or on human basophils. Very few reports addressed eosinophil response to eCBs and in particular to 2-AG. Concerning mast cells, PEA is the most extensively investigated eCB (especially in wild-type rats) also due to the fact that these cells produce high levels of PEA and express both CB_1_ and CB_2_.^1,19^

#### AEA and innate immunity

The first evidence for an immunoregulatory role of eCBs on monocytes/macrophages came from a study on mouse alveolar macrophages, where AEA inhibited macrophage-mediated killing of tumor necrosis factor (TNF)-sensitive cells.^20^ Later evidence supported the anti-inflammatory activity of AEA, whereby it inhibited expression of proinflammatory mediators such as nitric oxide and interleukins, IL-6, IL-12, and IL23, and enhanced anti-inflammatory mediators such as IL-10 and CD200R.^1,21^ These immunosuppressive effects were mostly mediated by CB_2_. The presence of the endocannabinoid system (ECS) on DCs was demonstrated for the first time by Di Marzo and coworkers (AEA, 2-AG, PEA, CB_1_, CB_2_, and FAAH), with 2-AG levels increasing significantly on activated human DCs.^22^ At the same time, it was found that high (micromolar) doses of AEA induce apoptosis in murine bone marrow-derived DCs, through both CB_1_ and CB_2_ receptors, providing a potential mechanism for eCB-mediated immunosuppression of immune cells.^23^ Interestingly, the efficacy of AEA depended on its rapid hydrolysis by FAAH, whose pharmacological inhibition led to reduced resistance to apoptosis. The involvement of CB_1_ and CB_2_ in determining DC responses was clearly elucidated by analyzing the phenotypic and functional profile of murine bone marrow-derived DCs from CB_1_^−/−^CB_2_^−/−^ mice. Indeed, deletion of both receptors exacerbated DC function by increasing their activation markers, MHC-I/II, CD80, and CD86, and by eliciting a more robust T-cell response.^24^ In contrast, another work reported that nanomolar and low micromolar doses of AEA before sensitization increased both the expression of murine DC costimulatory molecules CD80/CD86 and IL-12/IL-23 production *ex vivo.*^25^ Moreover, our group investigated more deeply the two subsets of DCs, that is, myeloid and plasmacytoid DCs. Notably, we found that only AEA was able to inhibit TNF-α, IL-12p40, and IL-6 from activated myeloid as well as TNF-α and IFN-α release from plasmacytoid DCs, both in a CB_2_-dependent mechanism.^26^ Furthermore, AEA-mediated immunosuppression of both DC subsets was also paralleled by a reduced ability of myeloid and plasmacytoid DCs to polarize naïve CD4 T cells into Th1 and Th17 lineages. AEA has been shown to inhibit neutrophil migration, and its levels positively correlated with their phagocytic capabilities^1,27^; yet, many studies consistently reported a failure of AEA to effectively inhibit superoxide and hydrogen peroxide production, thus being almost ineffective in regulating microbial neutrophil burst reaction. Interestingly, these effects seemed to be independent of cannabinoid receptors. In addition, AEA has been shown to limit excessive mast cell maturation and activation in a CB_1_-dependent mechanism in a human hair follicle organ culture model, suggesting that normal skin mast cells are indeed modulated by the ECS.^28^ The involvement of AEA and CB_1_ in modulating human mast cell functions was further confirmed by the observation that in human airway mucosal mast cells, maturation and excessive activation were inhibited by the eCB tone through CB_1_ stimulation.^29^ A very recent and interesting work further unraveled the biological implication of AEA-CB_1_-mediated mast cell modulation in mast cell-deficient mice, showing that AEA activation of CB_1_ in mast cells induced MCP-1-mediated recruitment of monocytic and anti-inflammatory myeloid-derived suppressor cells.^30^

#### 2-AG in innate immunity

Controversial data exist on the role of 2-AG in the modulation of macrophages/microglia responses. On the one hand, 2-AG inhibits TNF-α and IL-6 production and promotes phagocytosis of opsonized zymosan and alternatively activated and anti-inflammatory M2 macrophages.^1^ On the other hand, it increases iNOS-dependent nitric oxide and chemokine production, as well as migration and cell adhesion.^1^ In some cases, it was not entirely clear whether the effects of 2-AG were actually mediated through CB_2_ receptors. Additionally, it has been suggested that discrepancies on the effects of 2-AG can be due to their conversion into bioactive COX-2 metabolites.^10^ On DCs, 2-AG acts as a chemoattractant for both immature and mature bone marrow-derived mouse DCs, also shifting the response toward the Th1 type.^31^ In parallel, 2-AG seems to be also an activator of human neutrophils by stimulating myeloperoxidase release, leukotriene B_4_ biosynthesis, kinase activation, and calcium mobilization.^32^ It also induces increased levels of antimicrobial effectors, thereby being a potent regulator of host defense *in vivo*. As expected, these effects on neutrophil activation were not mediated by CB_2_, also due to the very low levels of its expression in these cells, but were rather due to its hydrolysis and subsequent metabolism into LTB_4_, with activation of BLT_1_ receptors. Additional data supported a role for 2-AG in controlling RhoA activation, thereby suppressing neutrophil migration.^33^ Almost no evidence exists on the effects of eCBs on NK cells, where this compound induced the migration of NK-differentiated human HL-60 cells through CB_2_.^1,34^ 2-AG induced the migration of human eosinophils in a CB_2_-dependent manner, where such receptor was particularly expressed, although less potently than typical, strong eosinophil chemoattractants such as platelet-activating factor, RANTES, and eotaxin.^35^ These studies suggest that CB_2_ and its endogenous ligand 2-AG may be potentially involved in allergic inflammation, accompanied by eosinophil infiltration, and this was demonstrated in a mouse model of contact dermatitis. A very recent article investigated the mechanisms of 2-AG-induced migration of human eosinophils, confirming that this eCB in combination with IL-5 has the ability to activate and modulate eosinophil functional responses and that the 15-LOX pathway is very likely involved in the regulation of these activities.^36^

#### PEA in innate immunity

The immunomodulatory role of PEA has been, so far, investigated only on monocytes/macrophages and on mast cells. In particular, PEA exerts anti-inflammatory properties on the macrophages of the brain (i.e., microglia), mainly by stimulating phagocytosis and clearance of pathogens and by increasing resistance to infection and microglial cell motility.^1^ On mast cells, PEA is a strong inhibitor of mast cell degranulation and activation, also contributing to reduce the severity of spinal cord trauma.^19^ Interestingly, a recent work hypothesized that the anti-nociceptive role of PEA in inducing relief in neuropathic pain correlates with its ability to modulate these cells.^37^

## Adaptive Immunity

### T Lymphocytes

T lymphocytes (or T cells) play a central role in cell-mediated immunity and comprise several subsets (Fig. 1), each with a distinct function, including CD4+ T helper cells (Th), CD8+ cytotoxic T cells, memory T cells, regulatory T cells, γδ T cells, and mucosal-associated invariant T cells (MAIT).^38^ Although, it is not known whether these specific cell subsets are capable to produce eCBs, their expression of cannabinoid receptors has been extensively investigated and it seems that T cells usually bear very low levels of both CB_1_ and CB_2_.^1,39^ However, we have demonstrated that CB_2_ significantly increases CD4+ and CD8+ human T cells when activated,^39^ supporting the view that these cells are indeed responsive to the effects of eCBs.

### B Lymphocytes

B lymphocytes (or B cells) are not only involved in the production of antibodies against antigens (humoral immunity) but they are also capable of acting as antigen-presenting cells (Fig. 1).^40^ Antibody-producing plasma cells are among the immune cells that express the highest levels of CB_2_, with human B cells expressing one transcript and mouse B cells expressing three transcripts, specifically selected during B-cell activation by lipopolysaccharide. However, most of the research has focused only on the use of phytocannabinoids and syntho-cannabinoids, rather than on eCBs, trying to understand the functional role of this receptor in B cells. Indeed, CB_2_ was identified as a crucial receptor for mouse B-cell differentiation since the end of the 90s as it was markedly expressed in mantle zones of secondary follicles and less in germinal centers and its expression was downregulated during B-cell differentiation. Furthermore, CB_2_ was found to be essential also for mouse B-cell subset formation and for retention of immature B cells in bone marrow sinusoids and in the splenic marginal zone. CB_2_ was also reported to mediate immunoglobulin class switching from IgM to IgE, suggesting that this CB receptor could have a crucial role in the generation of B-cell repertoire and the regulation of Th2-type humoral responses.^1^

#### AEA in adaptive immunity

The first evidence for an immunosuppressive role of eCBs on T cells came just 2 years after the isolation and purification of AEA, demonstrating its dose-dependent antiproliferative effects on human T cells. Indeed, micromolar doses of AEA rapidly inhibited mitogen-induced DNA synthesis and this was associated with induction of apoptotic cell death.^1^ Since then, most of the literature focused only on phytocannabinoids and synthetic agonists/antagonists selective for CB_1_ or CB_2_. It is now well accepted that AEA is a potent immunosuppressor of T-cell proliferation and cytokine release, acting mainly through CB_2_ and PPAR-γ and most likely through NF-kB inhibition. This pathway has been largely investigated in mouse and human T cells.^1,41,42^ Our group was the first to demonstrate the antiproliferative effect of AEA on both CD4 and CD8 T-cell subsets, without any effect on cell viability.^39^ In addition, we disclosed its inhibitory effect on IFN-γ-producing Th1 and IL-17-producing Th17. This effect of AEA on Th17 has been recently reproduced in a mouse model of hypersensitivity, where it was also shown to be mediated by IL-10 and miRNA induction.^43^ Interestingly, cytokines have been shown to directly influence other elements of the eCB system in T lymphocytes since Th2 cytokines, IL-4 or IL-10, had a stimulatory effect on FAAH, whereas the Th1 cytokines, IL-12 and IFN-γ, reduced FAAH activity and protein expression, overall suggesting an eCB-triggered self-sustaining anti-inflammatory loop. The strong involvement of CB_2_ in mediating AEA anti-inflammatory effects is supported by a reduction of eCB immune modulation of T cells from a common CB_2_ polymorphism and the evidence that formation of T cells requires this receptor.^1^ Surprisingly, no evidence of AEA has been gathered so far in either mouse or human B cells.

#### 2-AG in adaptive immunity

Of note, only two works of Rockwell et al. demonstrated directly the effect of 2-AG on T lymphocytes, where it induced a significant suppression of IL-2 expression^44^ and such anti-inflammatory effect was independent of CB receptors, but it was rather mediated by a COX-2 metabolite of 2-AG, probably by activating the PPAR-γ.^45^ As for B lymphocytes, this bioactive lipid induced migration of B220+CD19+ B cells, preferentially by attracting unstimulated naïve B cells rather than activated and/or class-switched germinal center B cells in a CB_2_-dependent manner.^46^ It has been postulated that these effects might be indirect since they could involve other immune cells (such as T cells and macrophages) that are required for B-cell activation.

## Conclusions

**FIG. 2.**
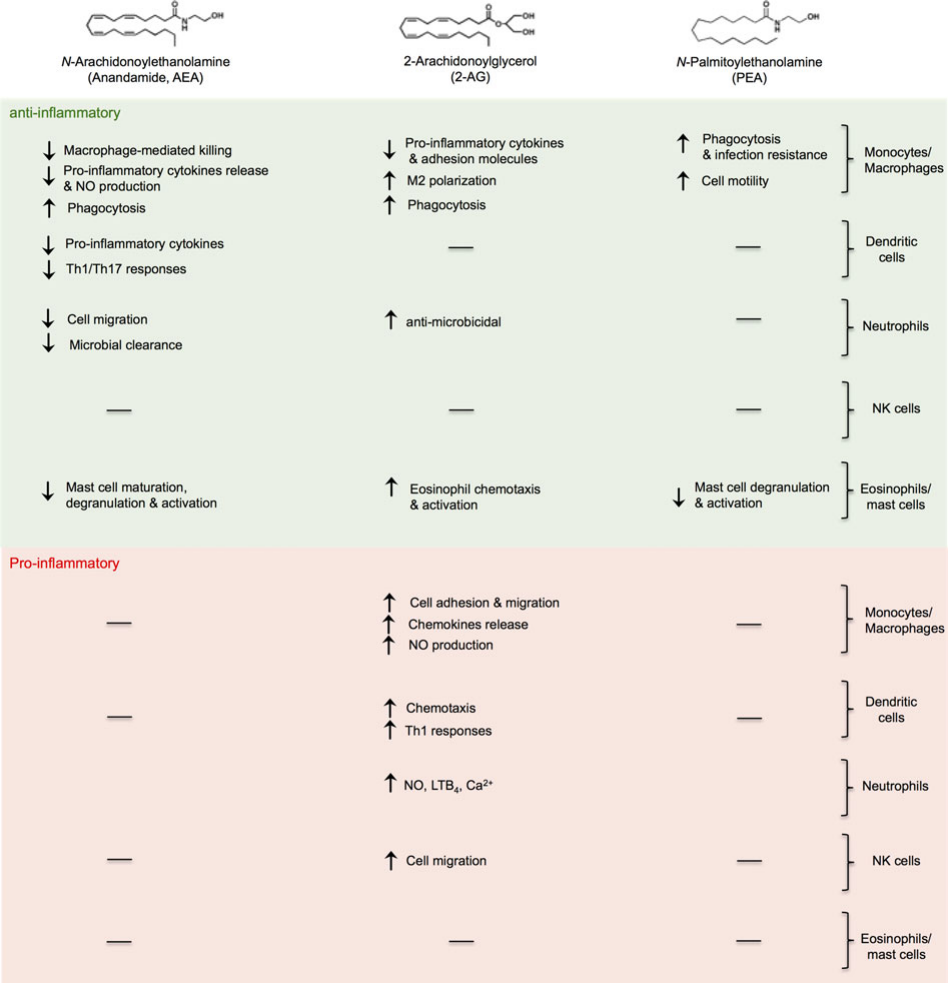
Anti-inflammatory and proinflammatory effects of eCBs on cells of innate immunity. eCB, endocannabinoid.

**FIG. 3.**
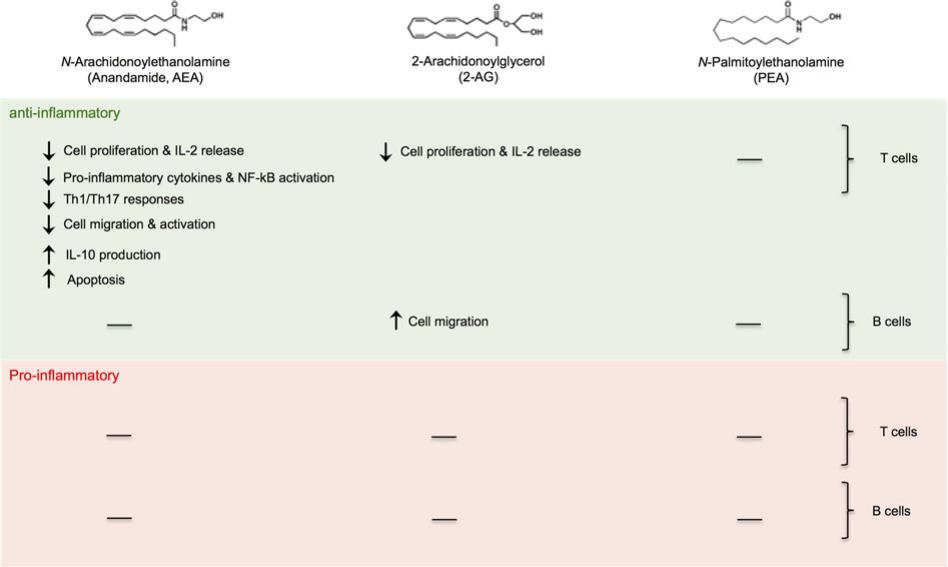
Anti-inflammatory and proinflammatory effects of eCBs on cells of adaptive immunity.

The majority of scientific studies on the immunoregulatory role of eCBs concentrated on whole immune cells, either on peripheral blood mononuclear cells or on mouse splenocytes. When investigating the specific immunoregulatory role of eCBs on each specific immune cell subset of both innate (Fig. 2) and adaptive (Fig. 3) immunity, most of the research has focused on monocytes/macrophages and T cells and mainly on AEA. Of note, AEA is the most potent anti-inflammatory eCB and it practically acts on all cell subsets of either innate or adaptive immunity (except for NK and B cells). Instead, 2-AG exerts both pro- and anti-inflammatory effects and it seems that its effects are strictly dependent on cell type. Although some specific and rarely represented immune cells (i.e., regulatory T cells, γδ T cells, or MAIT cells) were never investigated, neither were the subpopulations of each innate immune cell type, it can be generally stated that eCBs, particularly AEA and PEA, could be considered as master regulators of the innate-adaptive immune axis and as valuable immunoresolvents. Indeed, the fact that most of these molecules as well as several elements of their metabolism and signaling (i.e., enzymes and receptors) are dysregulated in many pathological states where the immune system is a crucial factor suggests that their exploitation in treating several chronic inflammatory diseases could just be around the corner, providing that their role will be also confirmed *in vivo* and that their underlying molecular mechanism elucidated.

## Abbreviations Used

2-AG: 2-arachidonoylglycerol
COX-2: cyclooxygenase-2
DC: dendritic cell
eCB: endocannabinoid
ECS: endocannabinoid system
FAAH: fatty acid amide hydrolase
ABDH4: α/β-hydrolase 4
LOX: lipoxygenase
MAIT: mucosal-associated invariant T cells
NAE: *N*-acylethanolamine
NAPE-PLD: *N*-acyl-phosphatidylethanolamine-hydrolyzing phospholipase D
NK: natural killer cells
OEA: *N*-oleoylethanolamine
PEA: *N*-palmitoylethanolamine
PPAR: peroxisome proliferator-activated receptor
TNF: tumor necrosis factor
